# Differentiation of Chinese rice wine(Huangjiu) from different aging status based on HS-SPME-GC–MS combined with near infrared

**DOI:** 10.1016/j.fochx.2025.102676

**Published:** 2025-06-20

**Authors:** Guangfa Xie, Junhao Xie, Dongsheng Qian, Lan Wang, Guochang Sun, Qingzhong Mao, Zhifang Yu, Mengsha Hu, Qi Peng

**Affiliations:** a Key Laboratory of Pollution Exposure and Health Intervention of Zhejiang Province, College of Biology and Environmental Engineering, Zhejiang Shuren University, Hangzhou 310015, Zhejiang, China; b National Engineering Research Center for Chinese CRW (Branch Center), School of Life and Environmental Sciences, Shaoxing University, 900 Chengnan Road, Shaoxing, 312000, Zhejiang, China; c Shaoxing Testing Institute of Quality and Technical Supervision, No.8 Huagong Street, Town of Lihai, Shangyu, Shaoxing, 312366, China; d Zhejiang Shaoxing Huangjiu Industry Innovation Service Complex, China Shaoxing Yellow Rice Wine Group Co., Ltd. Shaoxing 312000, Zhejiang, China; e Kuaijishan Shaoxing Rice Wine Co., Ltd, Shaoxing 312000, Zhejiang, China

**Keywords:** HS-SPME-GC–MS, Chinese rice wine, Near-infrared, Chinese rice wine aging process, Principal component analysis

## Abstract

This study investigates the crucial task of determining the aging state of Huangjiu, which is vital for its commercial and scientific significance due to the development of sensory characteristics during aging. Focused on Huangjiu from Guyue Longshan winery, China's top producer, the analysis employed a near-infrared spectrometer and gas chromatography–mass spectrometry coupled with headspace-solid phase microextraction. Stoichiometric analysis interpreted the data, enabling prediction and classification of samples aged for 1, 5, 8, 10, and 20 years. Principal component analysis (PCA) and metabolite screening revealed systematic differences among the samples. Factor analysis and partial least squares regression analysis constructed a sublibrary model with an identification accuracy exceeding 95 %, PLS model's R^2^ surpassing 98 %, and RPD exceeding 5, facilitating precise sample year identification. These findings enhance understanding of the Huangjiu aging process and provide crucial technical support for maintaining the quality of Shaoxing Huangjiu, a product protected by geographical indication.

## Highlights

• Utilizing NIRS and HS-SPME-GC–MS for swift discrimination of rice wine of different ages.
• Identifying 148 volatiles, notably esters, aldehydes, alcohols, and acids, as robust markers.
• Alcohol and ester flavors are pivotal in age differentiation.

## 1 Introduction

Huangjiu, a traditional Chinese rice wine, is well-known for its complex aroma, rich colour, and mellow taste(Gao et al., 2024; Hu et al., 2023; Xie et al., 2021). Its flavor profile is influenced by multiple factors, including raw materials, fermentation processes, microbial communities, storage conditions, and, importantly, aging time (Feng et al., 2020; Peng, Zheng, Meng, Yu, et al., 2022; J. T. Yu et al., 2023). Among these, aging is a critical stage in quality enhancement, during which a series of physicochemical and biochemical transformations significantly alter the composition of volatile and non-volatile compounds (Peng et al., 2017; Yang et al., 2022). Studies have shown that prolonged aging leads to an increase in esters, aldehydes, organic acids, and Maillard reaction products, while reducing free amino nitrogen and certain alcohols(Cao et al., 2010; Gonen et al., 2021). These changes contribute to the development of a smoother mouthfeel and the emergence of layered aroma notes such as nutty, caramel, floral, and aged characteristics. For example, ethyl esters (e.g., diethyl succinate, ethyl lactate) and phenylethanol have been found to increase with storage time and are considered key contributors to the mature aroma of aged Huangjiu (Wang et al., 2023; H. Y. Yu et al., 2021). However, the variation in aroma-active compounds across different aging stages remains poorly characterized due to the lack of comprehensive analytical frameworks(Guo et al., 2024).

Accurate identification of Huangjiu's aging status is essential not only for quality control but also for preventing market fraud related to counterfeit and blended products (Sun et al., 2022). Traditional methods such as sensory evaluation are limited by subjectivity, while chemical fingerprinting using chromatography or spectroscopy, when combined with chemometric modeling(Wan et al., 2012), provides more objective and reproducible results (Fei Shen et al., 2010; Ziólkowska et al., 2016). Near-infrared (NIR) spectroscopy, in particular, is a rapid, non-destructive, and cost-effective technique that has been widely adopted in food and beverage quality assessment(Liu et al., 2023) but is still underutilized in the Huangjiu industry (Anjos et al., 2020; Wu et al., 2015). Meanwhile, headspace solid-phase microextraction coupled with gas chromatography–mass spectrometry (HS-SPME-GC–MS) has proven effective for profiling volatile compounds in complex matrices. It allows for the identification of aroma markers and the elucidation of age-related changes in chemical composition (H. Y. Yu et al., 2022). Nevertheless, the integration of NIR and HS-SPME-GC–MS, along with multivariate statistical modeling, remains a relatively unexplored approach for distinguishing Huangjiu samples by vintage.

In this study, we applied HS-SPME-GC–MS and NIR spectroscopy, coupled with chemometric analyses, to investigate the volatile compound profiles of semi-dry Huangjiu aged for 1, 5, 8, 10, and 20 years. By combining molecular-level detection with multivariate modeling, we aimed to construct a robust classification framework for vintage discrimination and provide insights into the aroma evolution mechanisms of aged Huangjiu.

## 2 Materials and methods

### 2.1 Sample preparation

The samples utilized in this study originated from five distinct groups of semi-dry Huangjiu (The sugar content ranged from 15.1 g/L to 40.0 g/L) exhibiting varying degrees of aging, sourced from Guyue Longshan Company (Table S1). These samples were meticulously stored at 4 °C, shielded from light, to maintain optimal conditions. Prior to analysis, each sample was housed in a dark, dry glass container to ensure the preservation of its original properties.

### 2.2 NIR Spectrum collection

To capture NIR spectra, each prepared sample was carefully positioned within a quartz sample cup (5 mm in diameter, 10 mm in height, capable of holding 1 g). Gentle compression was applied to uniformly flatten the sample surface. A Fourier near-infrared spectrometer (MPA model by Bruker Company, Germany), equipped with a diffusion integrating sphere and a GaAs detector, was then utilized. The absorption mode was employed, covering a range from 13,000 to 4000 cm^−1^, with a resolution of 8 cm^−1^. Spectral acquisition utilized the instrument's built-in gold plating, and OPUS 7.5 software facilitated the collection of spectra for each sample. An average spectrum was generated through 32 scans to ensure data reliability and accuracy.

### 2.3 NIR spectral analysis

For model calibration, 80 % of the total samples were used, while the remaining 20 % were set aside for validation, following the methodology detailed by(F. Shen, Yang, Ying, Li, Zheng, & Jiang, 2012). Ensuring robust model training, two samples from each group were randomly chosen for the prediction set, with the remainder allocated to the correction set. Preprocessing of the spectral dataset included mitigating baseline dispersion, random noise through smoothing and first-order (1D) corrections, and addressing particle size effects via elimination of constant offset and vector normalization (SNV). The selection of the optimal wavenumber range aimed to minimize the influence of unrelated variables.The preprocessed spectral variables were subsequently selected for both classification identification and model quantification. The entire spectrum underwent even subdivision into subintervals, with a Partial Least Squares (PLS) regression model established for each subinterval. Extensive testing of spectral preprocessing methods and wavenumber ranges, conducted individually and in combination, aimed to pinpoint the most effective sub-range, thereby enhancing the calibration model's performance. Utilizing the Factor Analysis (FA) method, as delineated(Peng, Zheng, Meng, Zhu, et al., 2022), facilitated the creation of the primary database distinguishing wine age (Table S2). Additionally, a sub-database was employed to construct the NIR spectral classification model specifically for JN10 and JN20 samples. The selective S-value is calculated as the distance between the average spectra of two groups of samples in the principal component space. It quantifies the extent to which the samples from different aging periods are distinguishable in the spectral domain, helping to evaluate the differentiation ability of the model.

Model performance evaluation relied on selective S-values($S=\frac{D}{\mathrm{DT}1+\mathrm{DT}2}$), which signify the distance between the average spectra of two Huangjiu samples. Qualitative analysis of spectral preprocessing methods and factorization models was facilitated using the Identity module in OPUS 7.5. Assessment of the PLS model involved statistical parameters such as the root mean square of the estimated error (RMSEE) and coefficient of determination (R^2^), as outlined by (Genisheva et al., 2018).Enhancing the PLS calibration models involved selecting the most relevant wavelengths, optimal preprocessing methods, and optimal modeling dimensions (Szigedi et al., 2012). Consequently, in quantitative procedures, the “dimension” parameter was employed to guide variable selection. All calculations were executed using OPUS 7.5.

### 2.4 GC–MS data collection and analysis

For GC/MS analysis, a 20 ml screw cap bottle was utilized, containing 1 ml of Huangjiu sample, 4 ml of pure water, 2 g of sodium chloride, and 10 μl of internal standard (IS) 2-O (68.344 ppm). The mixture was prepared for volatile compound extraction and absorption using a polyacrylate fiber (50 μm divinylbenzene (DVB)/carboxen (CAR)/polydimethylsiloxane (PDMS)) inserted into the bottle. Extraction occurred at 50 °C for 40 min. The gas chromatograph (GC) coupled with a mass spectrometer (MS) (SCION SQ-456; Bruker Dal tonics Inc., USA) utilized a DB-Nylon Wax column (60 m × 0.25 mm × 0.25 μm, Agilent Technologies, USA). Nitrogen served as the carrier gas at a flow rate of 2.0 ml/min. After injection, the fibers desorbed aromatic compounds for 4 min at 250 °C. The heating program involved initial conditions of 35 °C for 3 min, followed by a gradual increase to 60 °C, further raised to 250 °C at a rate of 3 °C/min, and maintained for 5 min. Mass spectrometry operated with electron bombardment ionization (EI) at an electron energy of 70 eV, an ion source temperature of 230 °C, and a scanning range of 30.00 to 350.00 amu (Chen et al., 2021).

Volatile compounds were identified and quantified using the internal standard method, with sec-octanol (final concentration: 1 mg/L) serving as the internal standard (S. Y. Li et al., 2020). The chemical composition and flavor profiles of each type of Huangjiu were analyzed, with each analysis repeated three times. The results are expressed as mean ± standard deviation (mg/L). Data from HS-SPME-GC–MS and chemical indicators were processed using SIMCA 14.1(MKS Data Analytics Solutions) and Microsoft Office Excel 2019 (Microsoft Corporation). Principal component analysis (PCA-X mode) in SIMCA, with UV (unit variance scaling) scaling (giving all variables equal importance), was employed to investigate the relationship between different brewing stages and the volatile flavor compounds in Huangjiu.(Peng, Zheng, Meng, Zhu, et al., 2022).

## 3 Result discussion

### 3.1 NIR Spectrum

**Fig. 1:**
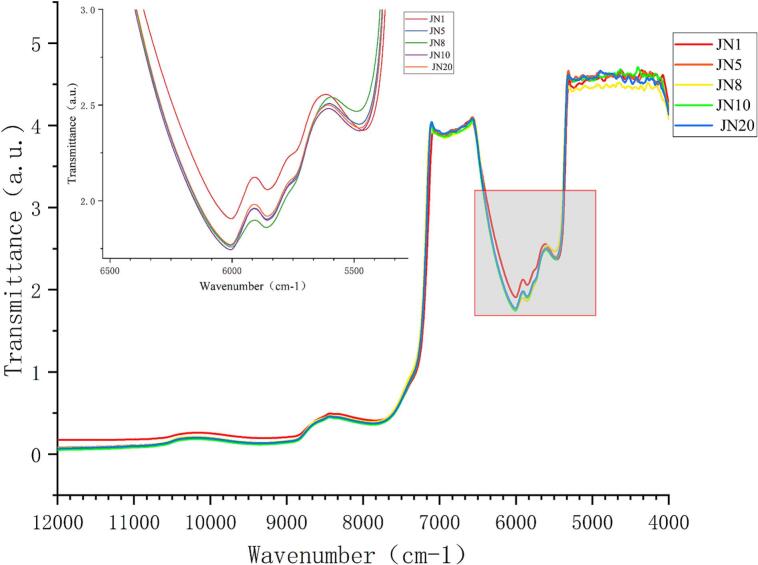
Average Spectrum of Huangjiu of Different Ages.

The average NIR spectra spanning the 10,000–4000 cm^−1^ range for JN1, JN5, JN8, JN10, and JN20 (Fig. 1). Notably, significant differences are evident between the spectra of the JN1 and JN8 groups, indicating variations in their chemical compositions. While absorption peak positions in JN5, JN10, and JN20 samples remain consistent, variations in peak intensities, particularly in the 10,500–9500 cm^−1^ range, suggest a potential association with the stretching vibration of N—H bonds. The intensity of the absorption band in this range may be linked to the second-order frequency doubling of protein N—H stretching vibrations. The peak around 8200 cm^−1^ is attributed to the second overtone C—H stretching of ethanol, a major Huangjiu component, and a combination of O—H bending, including the first overtone of O—H stretching influenced by water. Carbohydrates also contribute to this region, where the second overtone in the C—H stretching region is observable, along with a minor fraction from the -CH₂ of the amide I band and amide II band associated with second-order overtones of C—H antisymmetric vibrations. Distinct bands include the 6000–5800 cm^−1^ band linked to the first-order frequency doubling absorption of N—H stretching vibrations and the 5500–5300 cm^−1^ band associated with combined frequency absorption of N—H stretching vibrations. Visual observation alone cannot effectively distinguish differences between samples due to the complex characteristics of overtone and absorption bands in NIR spectral data. Hence, current research direction emphasizes developing sample recognition models based on chemometrics to extract meaningful insights from this intricate spectral information.

### 3.2 Analysis of NIR Huangjiu vintage qualitative model

**Fig. 2:**
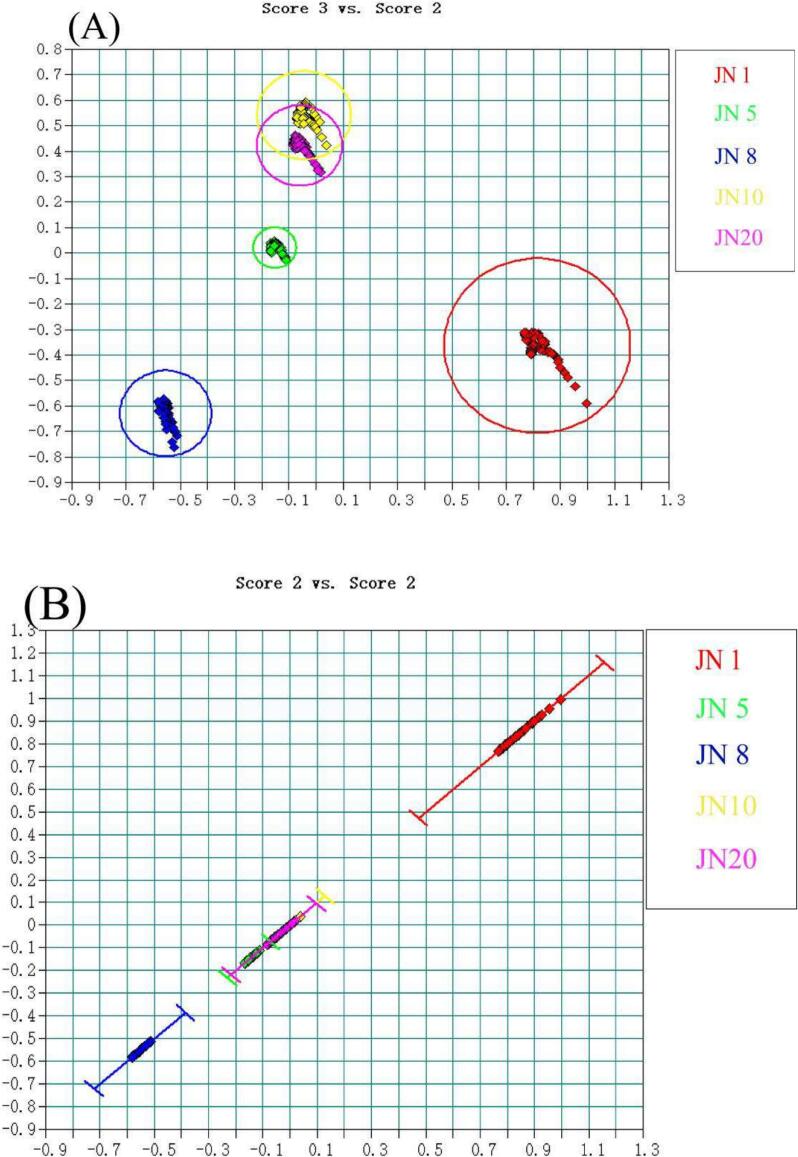
2D Score Chart Factorization Method (A) Level IP-1, (B) Level IP-1, (C) Level IP-2. Note: JN 1 - Red; JN 5 - Green; JN 8 - Blue; JN 10 - Yellow; JN 20 – purple **(D)**Principal Component Analysis (PCA) of the NIR spectra in Five Groups of Rice Wines at Different Vintage Stages. (For interpretation of the references to colour in this figure legend, the reader is referred to the web version of this article.)

Factor Analysis (FA) serves as a pivotal tool for identifying Huangjiu vintage through sample classification. The refinement of the model involves the meticulous selection of variables to assess the wavenumber range and the feasibility of the pretreatment method. In the initial step, the primary database undergoes preprocessing within the range of 6198.1–5380.7 cm^−1^, employing Standard Normal Variate (SNV). The calibration set comprises 360 spectra, while an additional 90 spectra constitute the test set. Employing two factors facilitates the effective differentiation of the five vintages of Huangjiu samples (Fig. 2A).Subsequently, a secondary sublibrary is established specifically for undistinguished samples. Spectra within the range of 6156 cm^−1^ to 6469.4 cm^−1^ remain unprocessed. The calibration set comprises 144 samples, with 36 samples allocated to the test set. This method effectively distinguishes between JN10 and JN20 by optimizing the number of factors (Fig. 2C). As illustrated in Fig. 2C, optimizing the number of factors allowed for a clearer separation between JN10 and JN20. This adjustment enhanced the model's ability to capture subtle spectral differences between these two closely aged samples, thereby improving the resolution and classification accuracy of the qualitative model.Remarkably, the FA model achieves a wine age prediction rate of 100 % across the five sample groups (Table S3).

### 3.3 NIR principal component analysis

Principal Component Analysis (PCA) serves as a powerful tool for data reduction and regularity analysis (Fearn, 2008). This technique involves matrix analysis, wherein feature vectors are standardized to reduce data dimensions and eliminate redundancy. Following the principle of variable elimination, where the principal component with the smallest eigenvalue corresponding to the largest eigenvector is iteratively removed, the remaining variables undergo principal component analysis. In our study, factor analysis (FA) was first used to identify the key sources of variation within the dataset. Based on these extracted factors, we then employed principal component analysis (PCA) to explore the relationships among the samples in a lower-dimensional space. The use of PCA in this context served as a complementary tool to visualize the distribution and clustering patterns of samples influenced by the underlying variation identified through FA.

The results of PCA (Fig. 2D) elucidate the extent of variation in the NIR spectra among different samples. The first and second principal components account for 58.6 % and 20.9 % of the variance, respectively. The attribution areas of various product groups exhibit a regular pattern in the distribution of differences. A shorter distance between the attribution regions of the sample groups suggests minor differences, whereas a greater distance indicates more significant disparities between the sample groups. Notably, JN10 and JN20, as well as JN5 and JN8, are grouped into the same zone, while JN1 forms a distinct zone. The classification of years into specific regions is accompanied by substantial distances between them，Samples from different years can be effectively distinguished. Points within each yearly group are distributed and concentrated within a certain range, affirming the repeatability and distinguishability of each year. The PCA results robustly demonstrate the model's efficacy in capturing the nuanced variations in the samples.

### 3.4 Quantitative analysis of NIR Huangjiu

The volatile flavor substances detected in Huangjiu, comprising 148 compounds analyzed via HS-SPME-GC–MS, were predominantly alcohols, esters, and aldehydes. Notably, 2-phenylethanol, Diisobutylcarbinol, Diethyl succinate, Adipic Acid Diisopropyl Ester, Benzaldehyde, and decanal account for the largest proportion in Huangjiu. To enhance discriminatory and predictive capabilities for Huangjiu of different years, a quantitative analysis model integrating near-infrared spectroscopy was developed. Selecting variables from the original spectral data, a Partial Least Squares (PLS) model with superior predictive power was established (Fig. 3), as evidenced by coefficients of determination (R^2^) and root mean square error of estimation (RMSEE) values detailed (Table S4). All compounds exhibited R^2^ values exceeding 0.97, underscoring the model's robustness. Moreover, RMSEE values for Diethyl succinate, Diisobutylcarbinol, Adipic Acid Diisopropyl Ester, decanal, Benzaldehyde, and Diethyl succinate were below 5 % of the average sample value, with 2-phenylethanol slightly exceeding 15 %. Additionally, residual prediction deviation (RPD) values were calculated, defined as the ratio of the population standard deviation (SD) of NIR prediction to the cross-validation standard error (SECV). As per(Fearn, 2008), an RPD value exceeding 3 is considered fair for screening, while surpassing 5 indicates good quality control (Genisheva et al., 2018). Encouragingly, all RPD values calculated in this study exceeded 3, affirming the model's efficacy in quantitative analysis and its suitability for quality control applications (See Fig. 3, Fig. 3, Fig. 3, Fig. 3, Fig. 3 and Table S.4).

**Fig. 3:**
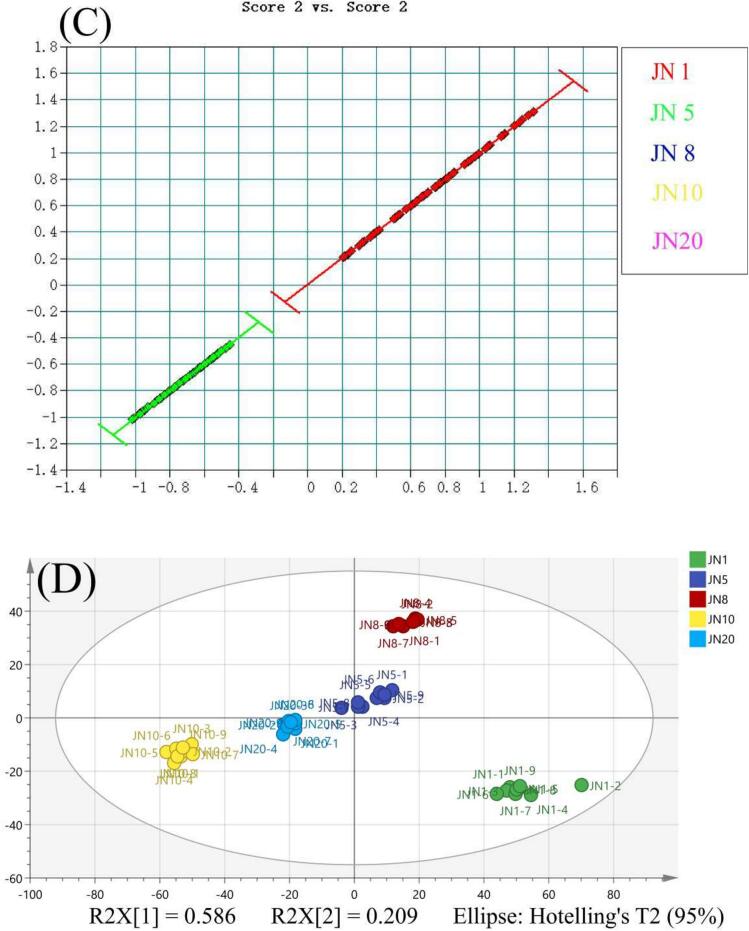
Partial least squares (PLS) regression results for 2-phenylethanol: (A1) Validation set; (B1) Calibration set. Note: Each point represents a Huangjiu sample from one of five aging stages (JN1, JN5, JN8, JN10, JN20).From left to right along the X-axis, and from lower to higher predicted concentrations, the samples correspond sequentially to JN1, JN5, JN8, JN10, and JN20.

**Fig. 3:**
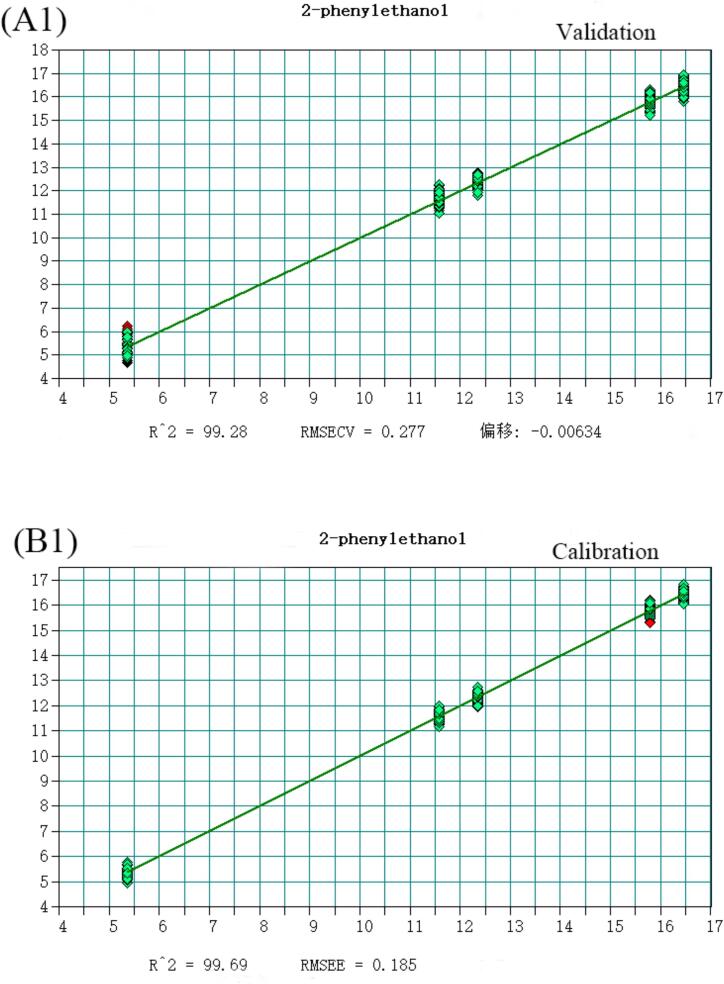
Partial least squares (PLS) regression results for Diethyl succinate: (A2) Validation set; (B2) Calibration set. Note: Each point represents a Huangjiu sample from one of five aging stages (JN1, JN5, JN8, JN10, JN20).From left to right along the X-axis, and from lower to higher predicted concentrations, the samples correspond sequentially to JN1, JN5, JN8, JN10, and JN20.

**Fig. 3:**
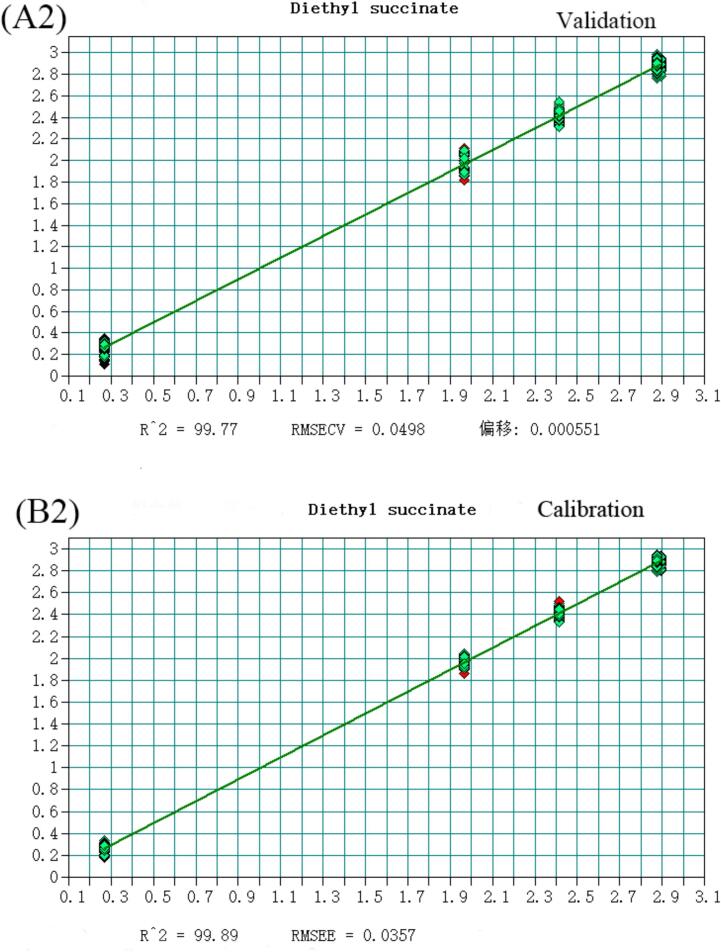
Partial least squares (PLS) regression results for Benzaldehyde: (A3) Validation set; (B3) Calibration set. Note: Each point represents a Huangjiu sample from one of five aging stages (JN1, JN5, JN8, JN10, JN20).From left to right along the X-axis, and from lower to higher predicted concentrations, the samples correspond sequentially to JN1, JN5, JN8, JN10, and JN20.

**Fig. 3:**
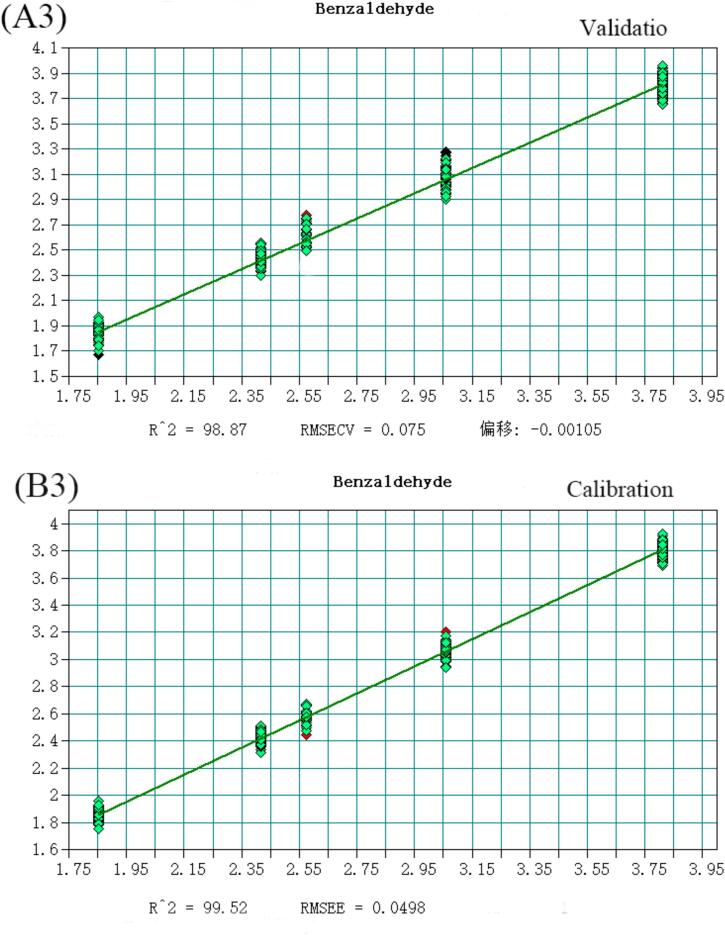
Partial least squares (PLS) regression results for Diisobutylcarbinol: (A4) Validation set; (B4) Calibration set. Note: Each point represents a Huangjiu sample from one of five aging stages (JN1, JN5, JN8, JN10, JN20).From left to right along the X-axis, and from lower to higher predicted concentrations, the samples correspond sequentially to JN1, JN5, JN8, JN10, and JN20.

**Fig. 3:**
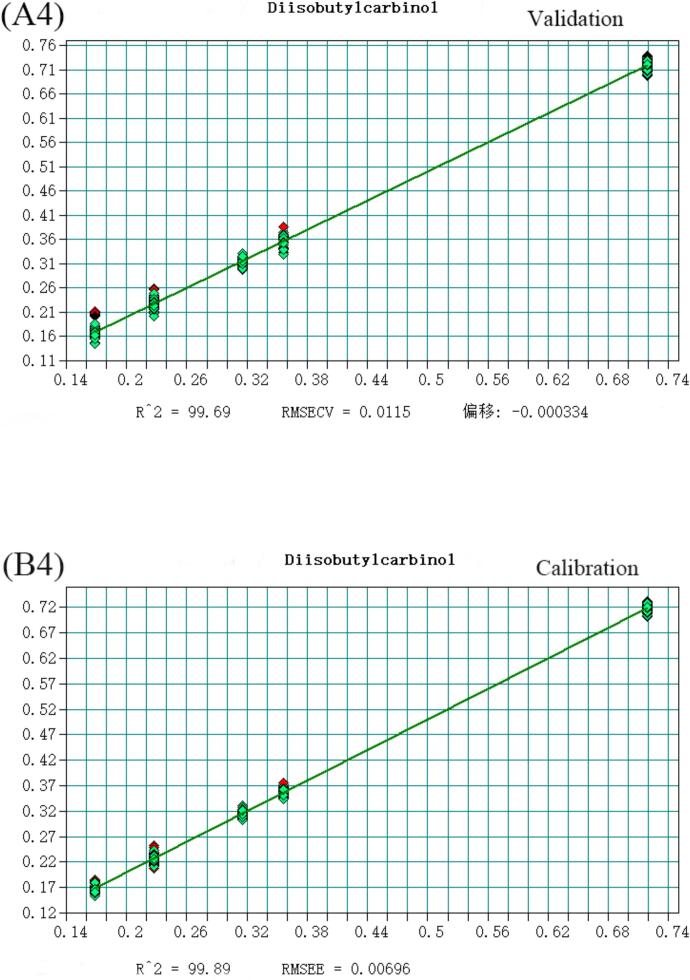
Partial least squares (PLS) regression results for Adipic Acid Diisopropyl Ester: (A5) Validation set; (B5) Calibration set. Note: Each point represents a Huangjiu sample from one of five aging stages (JN1, JN5, JN8, JN10, JN20). From left to right along the X-axis, and from lower to higher predicted concentrations, the samples correspond sequentially to JN1, JN5, JN8, JN10, and JN20.

**Fig. 3:**
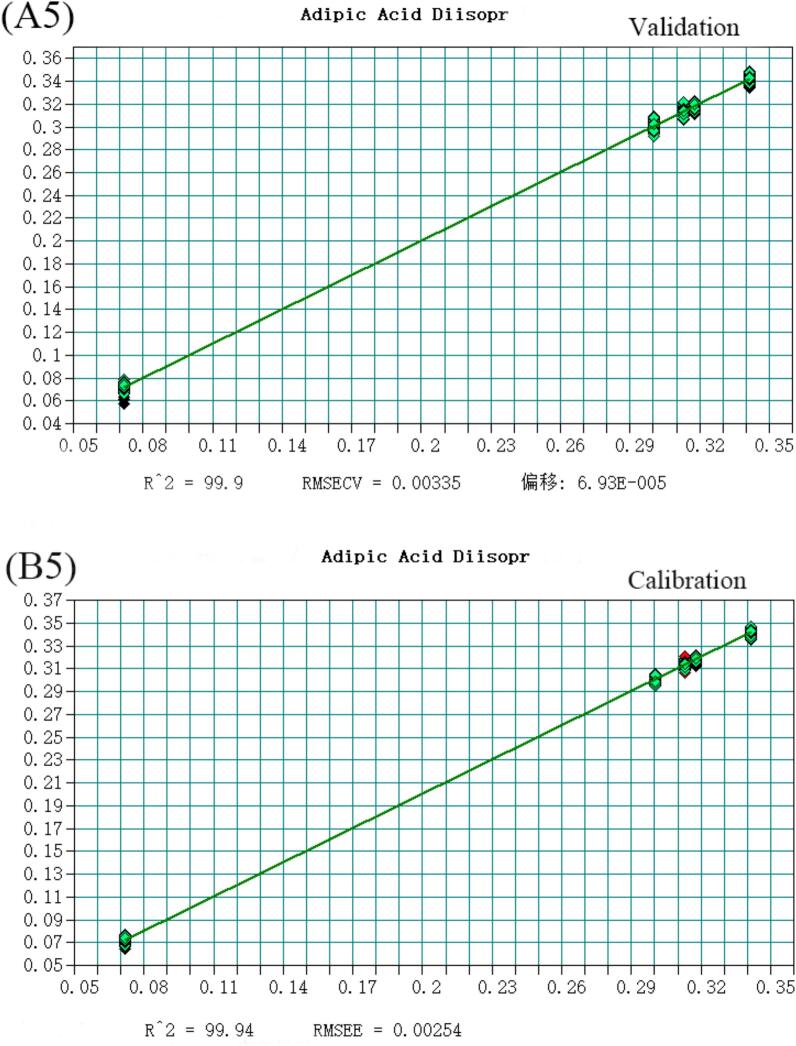
Partial least squares (PLS) regression results for Decanal: (A6) Validation set; (B6) Calibration set. Note: Each point represents a Huangjiu sample from one of five aging stages (JN1, JN5, JN8, JN10, JN20). From left to right along the X-axis, and from lower to higher predicted concentrations, the samples correspond sequentially to JN1, JN5, JN8, JN10, and JN20.

**Fig. 3:**
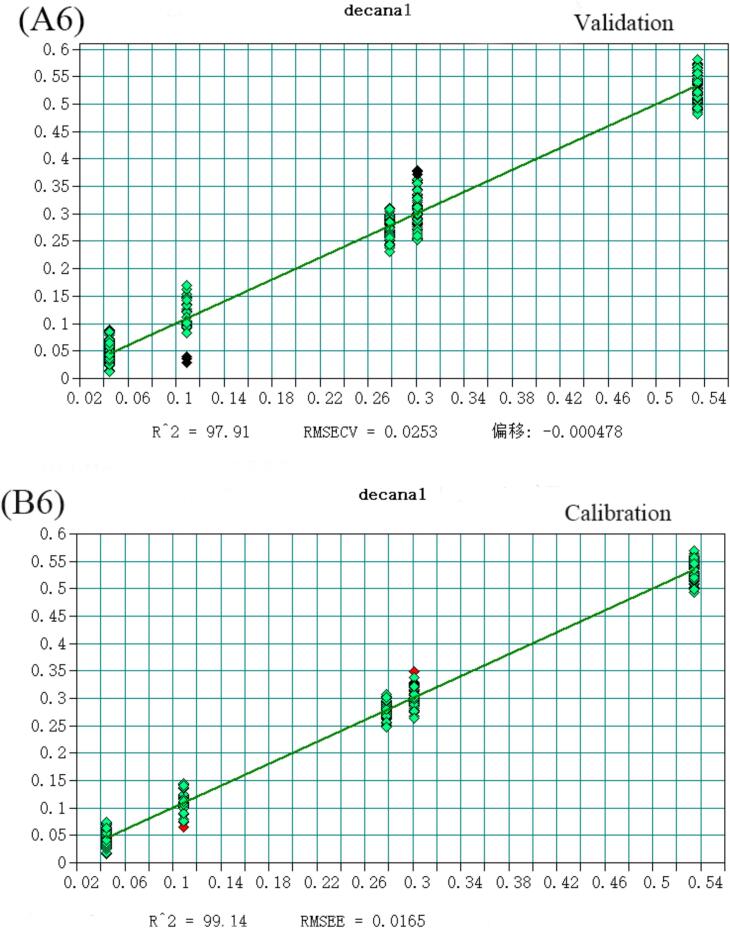
PLS Quantitative Maps for Different Years (A) Inspection, (B) Calibration.

### 3.5 Analysis of volatile compounds in Huangjiu of different vintages

The identification of 148 volatile compounds across 24 Huangjiu samples, categorized into 44 esters, 28 alcohols, 26 aldehydes, 8 acids, 6 ketones, 1 volatile phenol, 31 hydrocarbons, and 2 miscellaneous compounds (Table S5). Notably, esters, aldehydes, alcohols, and acids emerged as the primary volatile components.

Huangjiu undergoes an aging process sealed in clay pots with soil, facilitating catalytic oxidation reactions on the inner ceramic surface. This process notably influences the content of certain esters such as ethyl acetate, isobutyl acetate, isoamyl formate, ethyl lactate, phenylethyl acetate, gamma-decalactone, and ethyl phenylacetate. Concentrations of these esters increase with wine age, emphasizing their role in Huangjiu aroma. Esters, primarily formed through enzymatic catalysis or chemical esterification of organic acids and alcohols, were represented by ethyl acetate and diethyl succinate. Ethyl acetate contributes a fruity, irritating, and astringent taste, while diethyl succinate adds a fruity and sweet taste, potentially crucial in Huangjiu aroma profile. Some ethyl esters, such as ethyl caproate, ethyl caprylate, ethyl carnitate, ethyl oleate, and ethyl linoleate, exhibited decreased content with aging, while excess fatty acid esters formed during yeast hydrolysis in aging(Antalick et al., 2014).Moreover, total aldehyde content increased with wine age, reflecting alcohol oxidation during aging, aligning with previous research trends (Cao et al., 2010).

Alcohol compounds constituted the largest proportion of volatile flavor substances, with 2-phenylethanol being the most prominent. Renowned for its rose honey-like fragrance, 2-phenylethanol influences fermentation products, with notable presence in JN5, JN8, JN10, and JN20 samples. Twenty-six aldehydes were identified, with Benzaldehyde exhibiting the highest concentration. Benzaldehyde, known for its sweet, fruity, nutty, and caramel-like odor, displayed elevated levels of Benzaldehyde. Additionally, 2-phenyl-2-crotonal, characterized by a green, powdery, and cocoa aroma, emerged as a significant component of Huangjiu. Eight major acidic compounds, including 2-ethylcaproic acid, nonanoic acid, octanoic acid, and decanoic acid, were identified in the Huangjiu samples.This is similar to the results measured by (Feng et al., 2020).

### 3.6 Heat maps of different metabolites of volatile substances through screening

**Fig. 4:**
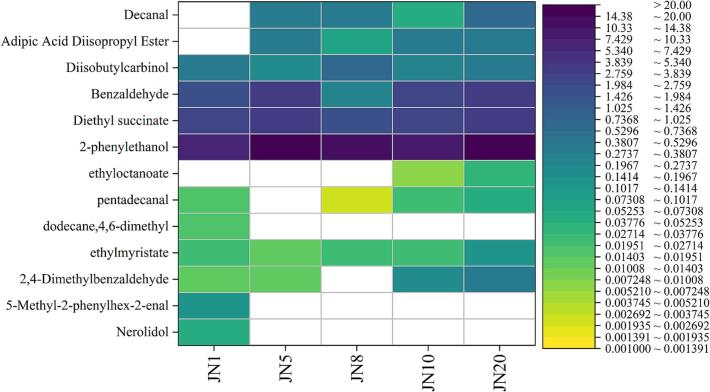
Comparative Heatmap of Key Volatile Compounds in Chinese Rice Wines Across Different Aging Years.

Differentially expressed volatile flavor compounds in Huangjiu were identified through multivariate statistical analysis. Compounds with VIP (Variable Importance in Projection) values greater than 1 and *p*-values less than 0.05 were selected as significantly differential metabolites. These compounds, along with other characteristic volatiles showing distinct abundance patterns, were used to construct the heatmap (Fig. 4), which visualizes the distribution and variation of key flavor compounds across Huangjiu samples with different aging periods. This comprehensive approach allows for the identification of volatiles contributing most significantly to aroma differentiation.

The heatmap revealed notable aging-associated variations in the concentration of specific compounds. For example, the relative abundance of 2-phenylethanol increased gradually with aging, indicating its accumulation over time, while decanal levels showed a decreasing trend. In the JN1 sample, 4,6-dimethyl-dodecane, 5-methyl-2-phenylhex-2-enal, and nerolidol were predominant, suggesting their possible role in the fresh aroma of young Huangjiu. Conversely, compounds such as 2,4-dimethylbenzaldehyde, ethyl myristate, pentadecanal, and ethyl octanoate were elevated in the JN20 sample, indicating their stronger influence on the mature aroma profile. These differences suggest that aging modulates the oxidative and esterification processes in Huangjiu, particularly enhancing aldehydes and esters in older vintages. The heatmap thus provides a clear visual summary of how key volatiles vary with aging and contribute to the evolution of Huangjiu's flavor characteristics (See Fig. 4)

### 3.7 Principal component analysis of Huangjiu in different years

**Fig. 5:**
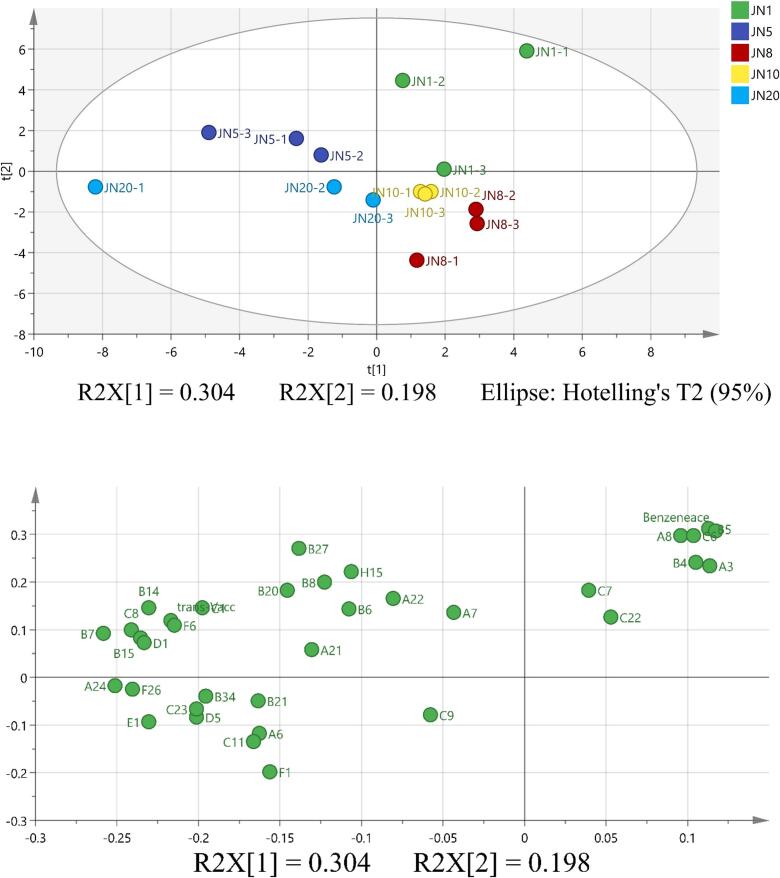
Biplot of Principal Component Analysis (PCA) Load and Score of Volatile Flavor Compounds in Five Groups of Rice Wines at Different Vintage Stages. Volatile Flavor Compounds and Codes are Defined in Table S5 and Table S1.

The flavor profile of Huangjiu is not an isolated entity but emerges through the intricate interaction of alcohol, ester, and aldehyde compounds (H. Y. Yu et al., 2021). To unravel the correlation between volatile flavor compounds and the various aging stages of five Huangjiu, a PCA was conducted (J. Li & Tao, 2013) using SIMCA 14.1. The PCA results (Fig. 5) unveil a robust relationship among volatile flavor compounds, with the first and second principal compounds (PCs) explaining 30.4 % and 19.8 % of the variance, respectively. This analysis highlights the distinctive distribution of points representing Huangjiu samples stored in bottles of different years. The regular positioning of points indicates that similar samples exhibit minor differences, while those situated farther apart reflect significant compositional disparities. The distribution of Huangjiu samples within their respective groups is concentrated within a defined range, underscoring the good repeatability of white wine samples stored in bottles of the same year. Notably, with the exception of JN8 and JN10, significant differentiation is observed among samples of Huangjiu stored in bottles of different years, each having its distinct region. This suggests that PCA substantial distance difference between JN1 and JN20 Huangjiu, indicating the most significant disparities in the types and contents of volatile substances between these two Huangjiu. The classification of years into specific regions is accompanied by substantial distances between them，Samples from different years can be effectively distinguished. The efficacy of PCA in determining the volatile flavor compounds in Huangjiu of different years, using headspace-solid phase microextraction and gas chromatography–mass spectrometry, supports its applicability in the identification and prediction of Huangjiu ages (See Fig. 5).

## 4 Conclusion

This study employed a comprehensive approach combining headspace-solid phase microextraction gas chromatography–mass spectrometry (HS-SPME-GC–MS) and near-infrared spectroscopy to investigate the volatile components of Huangjiu across different aging periods. Analysis identified 148 volatile compounds in five samples representing various wine ages, with esters, aldehydes, alcohols, and acids as primary volatile compounds. These findings are consistent with those reported by(Feng et al., 2020) regarding the aroma compounds in Huangjiu of different ages. HS-SPME-GC–MS analysis revealed nofih differences in alcohol and ester quantities and content among Huangjiu of different ages, providing characteristic markers for age differentiation. Referencing the potential volatile compounds identified in wine using NIR by (Genisheva et al., 2018), the selected potential volatile flavor compounds in Huangjiu were distinguished using factor analysis (FA). The partial least squares (PLS) model achieved an average R^2^ of 98.35 % and an RMSECV of 12.86 %, indicating high accuracy. The application of principal component analysis (PCA) effectively managed the numerous correlations among volatile organic compounds, enhancing result clarity by reducing data dimensionality and computational load.

Huangjiu is increasingly gaining popularity, particularly those aged for several years, which have garnered significant attention. Consequently, ensuring the consistent quality of Huangjiu is of paramount importance. This study utilizes a combined approach of headspace solid-phase microextraction gas chromatography–mass spectrometry (HS-SPME-GC–MS) and near-infrared (NIR) spectroscopy to monitor flavor changes during the aging process. By analyzing these changes, the study aims to refine production techniques and optimize the environmental conditions during aging to enhance the overall quality of Huangjiu. Through the integration of near-infrared spectroscopy, HS-SPME-GC–MS technology, and PCA data processing, this study achieved rapid and accurate identification of five groups of Huangjiu with distinct storage years. The combined approach presents a robust methodology for the efficient analysis and differentiation of Huangjiu based on aging characteristics.

## CRediT authorship contribution statement

**Guangfa Xie:** Writing – review & editing, Project administration, Funding acquisition. **Junhao Xie:** Writing – original draft, Methodology, Investigation, Data curation. **Dongsheng Qian:** Supervision, Conceptualization. **Lan Wang:** Investigation, Data curation. **Guochang Sun:** Investigation, Data curation. **Qingzhong Mao:** Supervision. **Zhifang Yu:** Data curation, Conceptualization. **Mengsha Hu:** Investigation, Conceptualization. **Qi Peng:** Investigation, Data curation.

## Declaration of competing interest

The authors declare that they have no known competing financial interests or personal relationships that could have appeared to influence the work reported in this paper.

## Data Availability

Data will be made available on request.

## Appendix A Supplementary data

Supplementary material: Near-infrared spectroscopy analysis results and flavor substance statistics Image 1
