# Supplementary material for: Differentiation of Chinese rice wine(Huangjiu) from different aging status based on HS-SPME-GC–MS combined with near infrared
Source: Food Chem X. 2025 Jun 20;29:102676. doi: 10.1016/j.fochx.2025.102676 (PMC12242002; doi:10.1016/j.fochx.2025.102676)
Supplement: Supplementary file 1 — Supplementary material: Near-infrared spectroscopy analysis results and flavor substance statistics [file mmc1.docx]

**Table S. 1 ：**Sample Grouping

| Age Classification | Types | Sample Codes |
| --- | --- | --- |
| 1-year | Semi-Dry | JN1 |
| 5-year | Semi-Dry | JN5 |
| 8-year | Semi-Dry | JN8 |
| 10-year | Semi-Dry | JN10 |
| 20-year | Semi-Dry | JN20 |

**Table S. 2** **：**Factor Analysis (FA) Method Main Library and Sub-Library Operational Result

|  | ID | Group 1 | Group 2 | IP-Level | S value | Threshold 1 (DT1) | Threshold 2 (DT2) | D |
| --- | --- | --- | --- | --- | --- | --- | --- | --- |
| Calibration set | 1 | JN1 | JN5 | IP1: 1 | 2.007511 | 0.352262 | 0.139653 | 0.987525 |
|  | 2 | JN5 | JN20 | IP1: 1 | 1.356319 | 0.139653 | 0.166013 | 0.414581 |
|  | 3 | JN8 | JN5 | IP1: 1 | 2.269337 | 0.204864 | 0.139653 | 0.781827 |
|  | 4 | JN10 | JN20 | IP2: 2 | 0.929851 | 1.004747 | 0.516232 | 1.414284 |
|  | 5 | JN20 | JN10 | IP2: 2 | 0.929704 | 0.516232 | 1.004747 | 1.414060 |

**Note:**

IP1: Differentiation among Huangjiu samples aged for 1, 5, 8, 10, and 20 years (JN1, JN5, JN8, JN10, JN20).

IP2: Focused differentiation between JN10 and JN20 samples.

S: The spectral distance between the average near-infrared spectra of two Huangjiu samples.

DT1: Threshold value corresponding to the first Huangjiu sample.

DT2: Threshold value corresponding to the second Huangjiu sample.

D: The calculated spectral distance between the two Huangjiu samples.

**Table. S3：**Prediction Results of the Factor Analysis (FA) Model for the 5 Sample Groups

|  | ID | Group name | Number of correct samples / total number of samples | Accuracy /% |
| --- | --- | --- | --- | --- |
| Prediction set | 1 | 1 | 18 / 18 (100%) | 100% |
|  | 2 | 5 | 18 / 18 (100%) | 100% |
|  | 3 | 8 | 18 / 18 (100%) | 100% |
|  | 4 | 10 | 18 / 18 (100%) | 100% |
|  | 5 | 20 | 18 / 18 (100%) | 100% |

**Table. S4** ：Partial Least Squares (PLS) Model Predicted Operational Results

| ID | Comp | R^2^ | RMSEE | RPD |
| --- | --- | --- | --- | --- |
| 1 | 2-phenylethanol | 99.69 | 0.185 | 17.9 |
| 2 | Diethyl succinate | 99.89 | 0.0357 | 29.5 |
| 3 | Benzaldehyde | 99.52 | 0.0498 | 14.4 |
| 4 | Diisobutylcarbinol | 99.89 | 0.00696 | 30 |
| 5 | Adipic Acid Diisopropyl Ester | 99.94 | 0.00254 | 42.5 |
| 6 | Decanal | 99.14 | 0.0165 | 10.8 |

**Table S. 5:** Volatile Flavor Substances in Huangjiu of Different Years Analyzed by HS-SPME-GC-MS (mg/L)

|  | Volatile matter |  | JN1 | JN5 | JN8 | JN10 | JN20 |
| --- | --- | --- | --- | --- | --- | --- | --- |
| Alcohols | A1 | 1-Hexanol | 0.0427±0.0739 | ND | ND | ND | ND |
|  | A2 | 1-Heptanol | 0.0164±0.0285 | 0.0198±0.0246 | ND | 0.021±0.0298 | ND |
|  | A3 | cedrol | 0.0207±0.019 | 0.0316±0.006 | 0.0484±0.0776 | 0.009±0.0127 | 0.0393±0.052 |
|  | A4 | 2-ethylhexan-1-ol | 0.0356±0.0528 | 0.0147±0.0186 | 0.004±0.0069 | 0.0074±0.0105 | ND |
|  | A5 | Diisobutylcarbinol | 0.3565±0.1977 | 0.1681±0.0359 | 0.7185±1.2109 | 0.2276±0.0217 | 0.3155±0.0658 |
|  | A6 | 1-Octanol | 0.0208±0.036 | ND | ND | ND | ND |
|  | A7 | 2-phenylethanol | 5.3699±2.4858 | 15.7874±5.32 | 12.3611±15.3344 | 10.272±0.1005 | 16.4567±3.5445 |
|  | A8 | nonan-1-ol | 0.0355±0.008 | 0.0466±0.0494 | ND | 0.044±0.0099 | 0.0384±0.036 |
|  | A9 | Nerolidol | 0.044±0.0283 | ND | ND | ND | ND |
|  | A10 | 1-Undecanol | 0.0562±0.0655 | ND | 0.004±0.0069 | ND | 0.0409±0.0709 |
|  | A11 | hexadecan-1-ol | 0.0516±0.0893 | 0.0507±0.0454 | 0.0279±0.0412 | ND | 0.0588±0.0625 |
|  | A12 | Trans-2-Undecen-1-ol | 0.0083±0.0144 | ND | ND | ND | ND |
|  | A13 | pentadecan-1-ol | 0.0122±0.0211 | ND | ND | 0.0061±0.0086 | 0.007±0.0122 |
|  | A14 | isoamylol | ND | 0.0417±0.0569 | ND | 0.0149±0.0211 | ND |
|  | A15 | 2-propylpentan-1-ol | ND | ND | ND | ND | ND |
|  | A16 | dodecan-1-ol | ND | 0.0904±0.0309 | 0.0142±0.0246 | 0.053±0.0125 | 0.0669±0.0606 |
|  | A17 | 1-Heptacosanol | ND | ND | ND | ND | ND |
|  | A18 | tetradecan-1-ol | ND | ND | ND | ND | 0.0264±0.0457 |
|  | A19 | Heptadecanol | ND | ND | ND | ND | ND |
|  | A20 | epi-cedrol | ND | 0.03±0.052 | ND | ND | ND |
|  | A21 | ethylene glycol monododecyl ether | ND | 0.0044±0.0076 | ND | ND | ND |
|  | A22 | 1-Docosanol | ND | 0.02±0.0204 | ND | ND | 0.0024±0.0042 |
|  | A23 | 2-octyldecan-1-ol | ND | 0.025±0.0433 | ND | ND | ND |
|  | A24 | octacosanal | ND | 0.0079±0.0138 | ND | ND | 0.0342±0.0381 |
|  | A25 | decan-1-ol | ND | ND | 0.0684±0.1185 | ND | ND |
|  | A26 | 1-HEPTADECANOL | ND | ND | ND | ND | ND |
|  | A27 | 2-hexyldecan-1-ol | ND | ND | ND | ND | ND |

**TableS. 5** (continued)

|  | Volatile matter |  | JN1 | JN5 | JN8 | JN10 | JN20 |
| --- | --- | --- | --- | --- | --- | --- | --- |
| Esters | B1 | 2,2,4-trimethyl-1,3-pentanediol diisobutyrate | 0.054±0.0493 | 0.0682±0.0591 | 0.0479±0.0536 | 0.0428±0.0198 | 0.1521±0.0662 |
|  | B2 | Ethyl lactate | 0.317±0.352 | 0.157±0.2718 | ND | ND | ND |
|  | B3 | isopropyl palmitate | 0.0548±0.0444 | 0.1075±0.0658 | 0.1596±0.2558 | 0.0059±0.0084 | 0.071±0.0449 |
|  | B4 | octan-2-yl hexadecanoate | 0.1364±0.2362 | ND | ND | ND | ND |
|  | B5 | bis(2-ethylhexyl) phthalate | 0.428±0.7412 | 0.037±0.064 | 0.0137±0.0237 | ND | 0.0088±0.0153 |
|  | B6 | Tributyl 2-acetylcitrate | 0.2939±0.2633 | 0.0412±0.052 | ND | 0.0083±0.0117 | ND |
|  | B7 | Adipic Acid Diisopropyl Ester | 0.3002±0.0569 | 0.3179±0.339 | 0.072±0.1247 | 0.3132±0.0406 | 0.3417±0.0595 |
|  | B8 | Ethyl nicotinate | 0.0729±0.053 | 0.3069±0.0704 | ND | 0.0115±0.0162 | 0.2214±0.2067 |
|  | B9 | Ethyl benzoate | 0.1393±0.0442 | 0.1787±0.0609 | ND | 0.1238±0.047 | 0.1792±0.0584 |
|  | B10 | Diethyl succinate | 2.5731±1.3765 | 3.0602±0.9858 | 1.854±2.6977 | 2.4148±0.1496 | 3.811±0.7389 |
|  | B11 | lactic acid isoamyl ester | 0.0045±0.0078 | 0.0525±0.0475 | ND | 0.0247±0.035 | 0.0306±0.0531 |
|  | B12 | Ethyl hexanoate | 0.0235±0.0407 | ND | ND | ND | ND |
|  | B13 | methyl salicylate | 0.1817±0.3146 | ND | 0.0309±0.0534 | ND | ND |
|  | B14 | 6-methylheptyl prop-2-enoate | 0.0041±0.0071 | ND | ND | ND | 0.0072±0.0125 |
|  | B15 | Phenylacetic acid ethyl ester | 0.1515±0.1419 | 0.3223±0.0711 | ND | 0.0648±0.0917 | 0.222±0.0421 |
|  | B16 | phenethyl acetate | 0.0474±0.0489 | 0.1733±0.0269 | 0.0085±0.0146 | 0.1016±0.0331 | 0.113±0.0332 |
|  | B17 | ethyl 3-hydroxyoctadecanoate | 0.0144±0.0249 | ND | ND | ND | ND |
|  | B18 | ethyl 3-phenylpropanoate | 0.0372±0.0398 | ND | ND | ND | ND |
|  | B19 | 1-O-ethyl 4-O-(3methylbutyl)butanedioate | 0.0571±0.0211 | 0.2661±0.104 | 0.0029±0.005 | 0.1106±0.0075 | 0.2549±0.0887 |
|  | B20 | diethyl octanedioate | 0.0557±0.0964 | ND | ND | ND | ND |
|  | B21 | diethyl nonanedioate | 0.2024±0.2494 | 0.1634±0.0577 | ND | 0.0192±0.0272 | 0.1671±0.0856 |
|  | B22 | ethyl myristate | 0.0232±0.0245 | 0.0105±0.0181 | 0.0204±0.0177 | 0.0196±0.0059 | 0.122±0.0355 |
|  | B23 | 2-Ethylhexyl salicylate | 0.0212±0.0368 | ND | ND | ND | ND |
|  | B24 | diisobutyl phthalate | 0.2111±0.311 | 0.4699±0.4121 | 1.9073±2.959 | 0.0884±0.0056 | 0.3402±0.3744 |
|  | B25 | ethyl hexadecanoate | 0.1145±0.1242 | 0.1608±0.1529 | 0.0693±0.062 | 0.0829±0.0302 | 0.2935±0.1442 |
|  | B26 | gamma-Nonanolactone | 0.1393±0.1294 | 0.2198±0.0687 | ND | 0.0686±0.0379 | 0.1673±0.1676 |
|  | B27 | Homosalate | 0.0195±0.0338 | ND | ND | ND | ND |
|  | B28 | dibutyl phthalate | 0.0161±0.0278 | 0.0637±0.1103 | 0.685±0.274 | 0.0503±0.0108 | 0.1629±0.1282 |
|  | B29 | l-ascorbyl dipalmitate | 0.0272±0.0472 | ND | 0.0209±0.0363 | 0.0427±0.0604 | ND |
|  | B30 | Methyl hexadecanoate | ND | ND | ND | ND | ND |
|  | B31 | cis-3-Hexenyl benzoate | ND | ND | ND | ND | ND |
|  | B32 | Butyl benzoate | ND | ND | ND | ND | ND |
|  | B33 | Ethyl Pentadecanoate | ND | ND | 0.0078±0.0136 | ND | 0.0259±0.0449 |
|  | B34 | 2-Ethylhexyl acrylate | ND | ND | ND | ND | ND |
| Esters | B35 | ethyl stearate | ND | 0.0038±0.0066 | 0.0033±0.0058 | ND | 0.0063±0.0065 |
|  | B36 | Isopropyl myristate | ND | ND | 0.0354±0.0612 | ND | 0.0114±0.013 |
|  | B37 | ethyl octanoate | ND | ND | ND | 0.0072±0.0101 | 0.0275±0.0198 |
|  | B38 | Ethyl butyrate | ND | ND | ND | ND | ND |
|  | B39 | Ethyl heptadecanoate | ND | ND | ND | ND | ND |
|  | B40 | n-octyl acrylate | ND | ND | ND | ND | ND |
|  | B41 | ethyl (2S)-lactate | ND | ND | ND | ND | 0.0059±0.0103 |
|  | B42 | trans-2-tridecenal | ND | ND | ND | ND | 0.0085±0.0148 |
|  | B43 | Diethyl glutarate | ND | ND | ND | ND | 0.0568±0.0984 |
|  | B44 | 4-Dodecanolide | ND | ND | ND | ND | 0.0423±0.0733 |

**TableS. 5** (continued)

|  | Volatile matter |  | JN1 | JN5 | JN8 | JN10 | JN20 |
| --- | --- | --- | --- | --- | --- | --- | --- |
| Aldehyde | C1 | Furfural | 0.5847±0.7444 | 0.368±0.0859 | ND | ND | ND |
|  | C2 | Benzaldehyde | 1.9654±0.6882 | 2.8956±0.6393 | 0.2662±0.3695 | 2.4161±0.1801 | 2.8778±2.5959 |
|  | C3 | phenylacetaldehyde | 0.0795±0.0765 | 0.0284±0.0492 | ND | ND | ND |
|  | C4 | Nonanal | 0.2058±0.1007 | 0.1687±0.0822 | 0.0407±0.038 | 0.0979±0.0364 | 0.3407±0.2888 |
|  | C5 | 2-phenyl-2-butenal | 0.2933±0.2792 | 0.4804±0.1428 | ND | 0.1503±0.0288 | 0.2234±0.068 |
|  | C6 | Tridecanal | 0.0119±0.0207 | ND | ND | ND | 0.0039±0.0067 |
|  | C7 | 5-Methyl-2-phenylhex-2-enal | 0.1377±0.0674 | ND | ND | ND | ND |
|  | C8 | 2-phenylprop-2-enal | 0.0056±0.0097 | ND | ND | ND | ND |
|  | C9 | trans-2-nonenal | 0.0032±0.0056 | 0.0141±0.013 | ND | ND | 0.0073±0.0127 |
|  | C10 | 2,4-Dimethylbenzaldehyde | 0.014±0.0122 | 0.0125±0.0216 | ND | 0.1428±0.186 | 0.3633±0.2425 |
|  | C11 | trans-2-tetradecenal | 0.046±0.0797 | ND | ND | ND | ND |
|  | C12 | dodecanal | 0.0281±0.026 | 0.0467±0.0283 | 0.0889±0.1305 | 0.0277±0.0176 | 0.1113±0.108 |
|  | C13 | tetradecanal | 0.0107±0.0107 | 0.0071±0.0123 | 0.004±0.0041 | ND | 0.019±0.033 |
|  | C14 | decanal | 0.1091±0.0951 | 0.3008±0.1338 | 0.278±0.3387 | 0.0448±0.0633 | 0.5343±0.5335 |
|  | C15 | 5,9,13-Trimethyl-4,8,12-tetradecatrienal | 0.0176±0.0305 | 0.0324±0.0562 | 0.3521±0.4593 | 0.0496±0.0258 | 0.1431±0.0789 |
|  | C16 | 13-methyltetradecanal | ND | ND | 0.0034±0.0059 | ND | 0.0743±0.1286 |
|  | C17 | trans-2-dodecenal | ND | 0.0809±0.1402 | ND | 0.0404±0.0572 | ND |
|  | C18 | 3-Heptylacrolein | ND | 0.0548±0.0949 | 0.0061±0.0106 | 0.0321±0.0454 | 0.0413±0.0379 |
|  | C19 | 2-Undecenal | ND | ND | ND | ND | ND |
|  | C20 | 4-propylbenzaldehyde | ND | 0.0393±0.0681 | ND | ND | ND |
|  | C21 | pentadecanal | 0.017±0.0207 | ND | 0.0021±0.0036 | 0.0217±0.0229 | 0.0511±0.0153 |
|  | C22 | icosanal | ND | ND | 0.0074±0.0128 | ND | ND |
| Aldehyde | C23 | Docosanal | ND | ND | 0.0023±0.004 | ND | ND |
|  | C24 | hexadecanal | 0.0089±0.0154 | ND | ND | ND | ND |
|  | C25 | octadecanal | ND | ND | ND | ND | ND |
|  | C26 | trans-2-tridecenal | ND | ND | ND | ND | 0.0085±0.0148 |

Table 5 (continued)

|  | Volatile matter |  | JN1 | JN5 | JN8 | JN10 | JN20 |
| --- | --- | --- | --- | --- | --- | --- | --- |
| Volatile acids | D1 | 2-Ethylcaproic acid | 0.0027±0.0047 | ND | ND | ND | ND |
|  | D2 | Nonanoic acid | 0.1439±0.125 | ND | ND | ND | 0.1549±0.2683 |
|  | D3 | Octanoic acid | 0.0053±0.0091 | 0.1052±0.1822 | ND | ND | 0.0822±0.1424 |
|  | D4 | decanoic acid | ND | 0.0506±0.0876 | ND | ND | 0.0753±0.0939 |
|  | D5 | Palmitic acid | ND | 0.5892±0.1424 | 0.0073±0.0126 | ND | 0.1972±0.2709 |
|  | D6 | tetradecanoic acid | ND | 0.4993±0.1893 | 0.3405±0.42 | ND | 0.2438±0.3579 |
|  | D7 | tridecanoic acid | ND | ND | ND | ND | ND |
|  | D8 | dodecanoic acid | ND | 0.0368±0.0638 | ND | ND | 0.1165±0.1676 |
|  | D9 | pentadecanoic acid | ND | ND | ND | ND | ND |
|  | D10 | n-Tridecanoic acid | ND | ND | ND | ND | ND |

**TableS. 5** (continued)

|  | Volatile matter |  | JN1 | JN5 | JN8 | JN10 | JN20 |
| --- | --- | --- | --- | --- | --- | --- | --- |
| ketone | E1 | 7,9-ditert-butyl-1-oxaspiro[4.5]deca-6,9-diene-2,8-dione | 0.1135±0.1139 | 0.1314±0.0331 | 0.0233±0.0258 | 0.043±0.0091 | 0.1058±0.0877 |
|  | E2 | 2-octanone | 0.0605±0.1048 | 0.0612±0.106 | 0.0682±0.1181 | 0.0823±0.1164 | ND |
|  | E3 | 2-Hydroxycyclopentadecanone | ND | 0.04±0.0693 | ND | ND | 0.0303±0.0524 |
|  | E4 | Phytone | ND | 0.0154±0.0266 | ND | ND | 0.0637±0.0693 |
|  | E5 | (-)-carvone | ND | ND | 0.0051±0.0089 | ND | ND |
|  | E6 | acetophenone | ND | ND | ND | ND | ND |

|  | Volatile matter |  | JN1 | JN5 | JN8 | JN10 | JN20 |
| --- | --- | --- | --- | --- | --- | --- | --- |
| hydrocarbon | F1 | dodecane,4,6-dimethyl | 0.016±0.012 | ND | ND | ND | ND |
|  | F2 | 2,3,5-Trimethyldecane | ND | ND | ND | ND | ND |
|  | F3 | Dodecane | 0.0303±0.0525 | 0.0347±0.0601 | 0.0737±0.1276 | ND | 0.0088±0.0152 |
|  | F4 | pentadecane | 0.0429±0.0605 | 0.0472±0.0445 | 0.3995±0.6511 | 0.0439±0.01 | 0.0457±0.0431 |
|  | F5 | Tetradecane | 0.1219±0.1474 | 0.0842±0.0974 | 0.2146±0.3436 | 0.007±0.01 | 0.0598±0.0337 |
|  | F6 | icosane | ND | 0.0435±0.0582 | 0.0159±0.0183 | ND | 0.0158±0.0138 |
|  | F7 | heptadecane | 0.0624±0.0697 | 0.1614±0.1023 | 0.0272±0.0366 | 0.069±0.0603 | 0.0854±0.1045 |
|  | F8 | henicosane | 0.1447±0.1936 | 0.2121±0.1533 | 0.393±0.5118 | 0.0796±0.023 | 0.2778±0.1843 |
|  | F9 | dotriacontane | 0.152±0.2633 | 0.2609±0.2493 | 0.0418±0.0496 | 0.0526±0.054 | 0.017±0.0295 |
|  | F10 | hexadecane | 0.0063±0.011 | 0.0199±0.0345 | ND | ND | 0.0138±0.0239 |
|  | F11 | Hentriacontane | ND | 0.0098±0.017 | 0.0041±0.007 | 0.0113±0.0159 | ND |
|  | F12 | 1-iododecane | ND | ND | 0.0839±0.1453 | ND | ND |
|  | F13 | isononacosane | ND | ND | 0.0021±0.0036 | ND | ND |
|  | F14 | 2-methylhexacosane | 0.0218±0.0244 | ND | 0.0054±0.0093 | 0.0038±0.0054 | 0.0062±0.0108 |
|  | F15 | pentacosane | 0.0039±0.0068 | ND | 0.0067±0.0116 | ND | ND |
|  | F16 | heptacosane | ND | ND | ND | 0.004±0.0056 | ND |
|  | F17 | n-Tetracontane | ND | ND | ND | 0.0061±0.0086 | ND |
|  | F18 | tridecane | ND | ND | ND | ND | ND |
|  | F19 | farnesane | 0.0066±0.0115 | ND | ND | ND | ND |
|  | F20 | nonadecane | ND | ND | ND | ND | ND |
|  | F21 | 2,6,10-trimethylpentadecane | ND | ND | ND | ND | 0.019±0.021 |
|  | F22 | 1,1-diethoxy-3-methylbutane | ND | ND | ND | ND | ND |
|  | F23 | 1-Methyl-3-propylcyclooctane | ND | ND | ND | ND | 0.0193±0.0334 |
|  | F24 | 7,11,15-trimethyl-3-methylidenehexadec-1-ene | 0.3005±0.5205 | ND | ND | ND | ND |
|  | F25 | 2-methyl-1-phenyl-1-butene | 0.0054±0.0094 | ND | ND | ND | ND |
|  | F26 | 14-Octadecenal | ND | 0.0166±0.0288 | ND | ND | ND |
|  | F27 | trans-2-undecenal | ND | ND | 0.0042±0.0073 | ND | ND |
|  | F28 | squalene | ND | ND | ND | ND | ND |
|  | F29 | (2,2-Diethoxyethyl)-Benzene | 0.0214±0.0239 | 0.0123±0.0214 | ND | 0.0087±0.0123 | ND |
|  | F30 | O-Xylene | ND | 0.1054±0.1825 | 0.0549±0.0951 | ND | 1.1924±2.0652 |
|  | F31 | Ethylbenzene | ND | ND | ND | ND | 0.0943±0.1634 |

**TableS. 5** (continued)

|  | Volatile matter |  | JN1 | JN5 | JN8 | JN10 | JN20 |
| --- | --- | --- | --- | --- | --- | --- | --- |
| Phenol | G1 | 2,4-Di-tert- butylphenol | 0.051±0.068 | 0.348±0.289 | 0.411±0.233 | 0.285±0.241 | 0.801±0.778 |

**TableS. 5** (continued)

|  | Volatile matter |  | JN1 | JN5 | JN8 | JN10 | JN20 |
| --- | --- | --- | --- | --- | --- | --- | --- |
| other | H1 | 2,6-Di-tert-butyl-p-benzoquinone | 0.0539±0.0687 | 0.0475±0.0823 | ND | ND | 0.0282±0.0488 |
|  | H2 | Caffeine | 0.3005±0.5205 | ND | ND | ND | ND |

**TableS. 5** (continued)

Note: The results are presented as the mean standard deviation of the three replicate ± of rice wine samples at different aging stages.

Abbreviation: ND, not detected.
